# Heart conduction system defects and sustained ventricular tachycardia complications in a patient with granulomatosis with polyangiitis. A case report and literature review

**DOI:** 10.5935/0103-507X.20170052

**Published:** 2017

**Authors:** Laryssa Passos Sarmento Santos, Victor Guerreiro Bomfim, Camila Fagundes Bezerra, Natália Vieira Costa, Rafael Barreto Paes de Carvalho, Ricardo Sobral de Carvalho, Rogério da Hora Passos, Olivia Carla Bomfim Boaventura, André Luiz Nunes Gobatto

**Affiliations:** 1 Hospital São Rafael - Salvador (BA), Brazil.; 2 Universidade Federal da Bahia - Salvador (BA), Brazil.; 3 Universidade de Salvador - Salvador (BA), Brazil.

**Keywords:** Granulomatosis with polyangiitis, Atrioventricular block, Heart conduction system, Pacemaker, artificial, Bradycardia, Case reports

## Abstract

Granulomatosis with polyangiitis is a rare systemic inflammatory disorder characterized by vasculitis of the small arteries, the arterioles and the capillaries together with necrotizing granulomatous lesions. This case reports on a young female patient, previously diagnosed with granulomatosis with polyangiitis, who was admitted to the intensive care unit with seizures and hemodynamic instability due to a complete atrioventricular heart block. The event was associated with multiple episodes of sustained ventricular tachycardia without any structural heart changes or electrolyte disturbances. In the intensive care unit, the patient was fitted with a provisory pacemaker, followed by immunosuppression with corticosteroids and immunobiological therapy, resulting in a total hemodynamic improvement. Severe conduction disorders in patients presenting granulomatosis with polyangiitis are rare but can contribute to increased morbidity. Early detection and specific intervention can prevent unfavorable outcomes, specifically in the intensive care unit.

## Abstract (pt)

A granulomatose com poliangiíte é um raro distúrbio inflamatório sistêmico que se caracteriza por vasculite de pequenas artérias, arteríolas e capilares, associada a lesões granulomatosas necrotizantes. Este artigo relata o caso de uma paciente com diagnóstico prévio de granulomatose com poliangiíte, admitida à unidade de terapia intensiva com quadro de crises convulsivas e instabilidade hemodinâmica em razão de bloqueio atrioventricular completo. Estas manifestações se associaram a múltiplos episódios de taquicardia ventricular sustentada; não havia alterações estruturais cardíacas, nem se detectaram distúrbios hidroeletrolíticos. Na unidade de terapia intensiva, a paciente foi submetida à implantação de marca-passo provisório, imunossupressão com uso de corticosteroides e terapia imunobiológica, resultando em melhora hemodinâmica completa. Distúrbios graves da condução cardíaca em pacientes com granulomatose com poliangiíte são raros, mas associam-se à grande morbidade. O reconhecimento precoce e o uso de intervenções específicas são capazes de prevenir a ocorrência de desfechos desfavoráveis, especialmente na unidade de terapia intensiva.

## INTRODUCTION

Granulomatosis with polyangiitis (also known as Wegener's granulomatosis) is a rare systemic inflammatory disorder of unknown etiology^(1,2)^ that is characterized by vasculitis of the small arteries, the arterioles and the capillaries as well as necrotizing granulomatous lesions and has a wide clinical presentation. The upper and lower respiratory tracts and the kidneys are the most commonly affected sites.^(1,3-5)^

Cardiac involvement has long been regarded as rare, but it can vary from a subclinical disease to a wide spectrum of abnormalities, including myocarditis, valvar lesions, conduction system defects, coronary arteritis and pericarditis.^(1,3)^

## CLINICAL CASE

**Figure 1:**
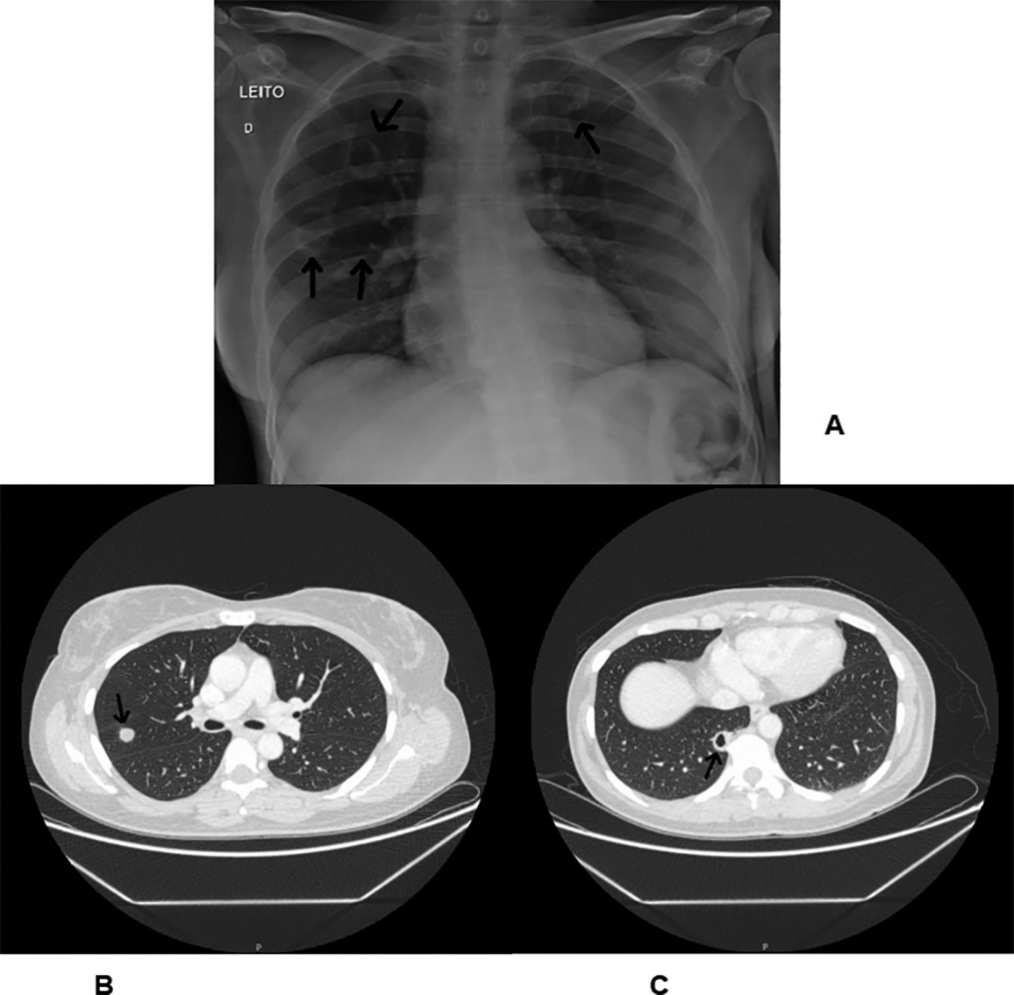
(A) Chest radiography in PA view showing bilateral nodules (patient previously diagnosed with Poliangiitis granulomatosis). (B and C) Same nodules showing thorax taken by computed tomography.

**Figure 2:**
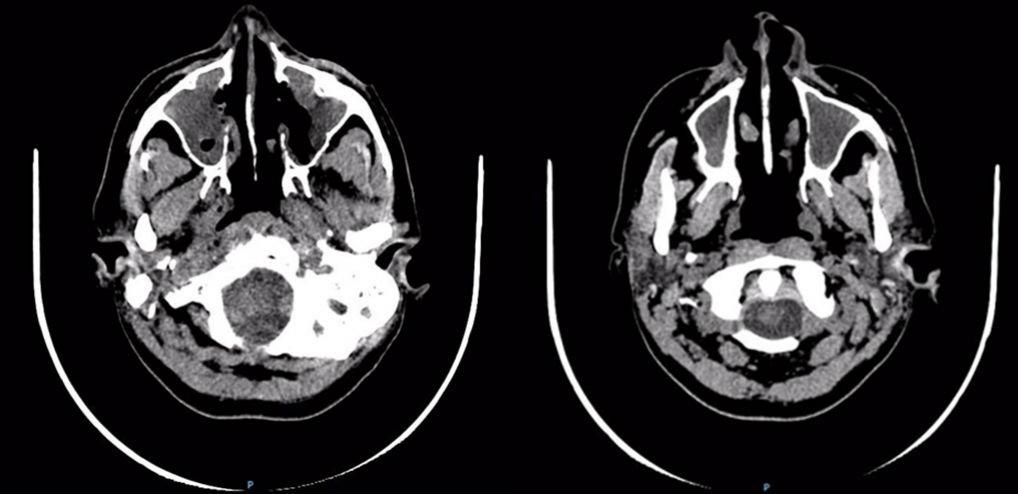
Cranial computed tomography in axial view showing an extensive compromise of the nasal cavity and maxillary sinus.

This report presents a case of a 44-year-old female patient diagnosed with granulomatosis with polyangiitis five months ago. The condition was characterized by involvement of the upper and lower respiratory tracts, a positive antineutrophil cytoplasmic antibodies (c-ANCA), a nasal mucosa biopsy, chronic unspecific inflammation, and no renal involvement. Bilateral nodules identified in the lung and a neck with imagem exams showed an irregular mucosal thickening of the paranasal sinuses, heavy padding of the bilateral ethmoid cells, partial absence of a turbinate, and preservation of the nasal septum along with a bilateral filling of the mastoid and the middle ear cells. Computed tomography (CT) of the skull and sinuses revealed an increased thickening of the maxillary and the sphenoid, a septum erosion, bilateral mastoiditis and pansinusitis (Figures 1 and 2). Thus, immunosuppressive therapy (cyclophosphamide and methylprednisolone) was administered, resulting in an unsatisfactory response to the treatment.

Over time, the symptoms did not improve, and the patient exhibited a worsening exophthalmia, drowsiness and apathy. The patient was admitted for hospitalization and started on an immunobiological therapy with rituximab. Overall, the patient's condition was poor. was dehydrated but did not exhibit signs of any respiratory or cardiac pathological abnormalities nor signs of an infection or metabolic or electrolyte disorders.

**Figure 3:**
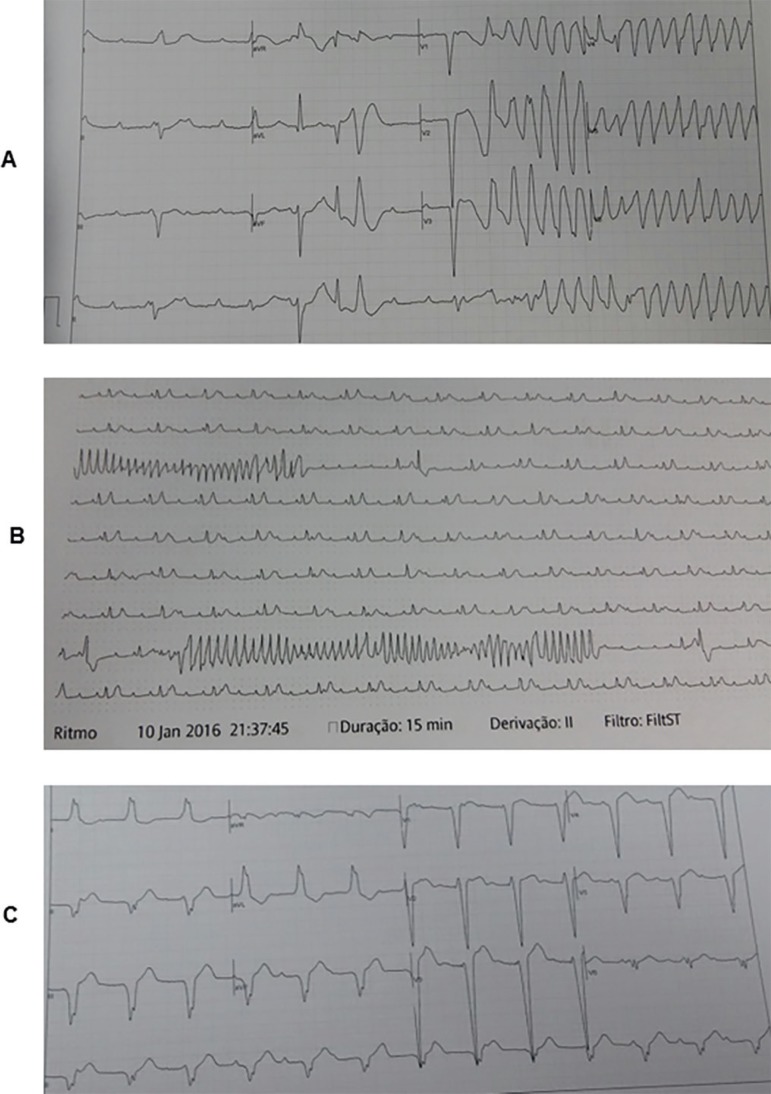
(A) Electrocardiogram showing a total atrioventricular block and a Torsades de points. (B) Multiple ventricular tachycardias in intensive care unit monitoring. (C) Same patient post-transvenous temporary pacing. We can see a complete atrioventricular dissociation.

On the first day of hospitalization, the patient presented with drowsiness followed by a tonic-clonic seizure episode that lasted roughly one minute. When the seizure subsided, she was bradycardic (32bpm) and hypotensive (84 x 48mmHg). The patient was immediately transferred to the cardiovascular intensive care unit, where a complete atrioventricular heart block was identified by bedside electrocardiogram, which was further associated with multiple episodes of sustained ventricular tachycardia (SVT) upon monitoring (Figure 3). The intensive care unit medical team performed a bedside transthoracic echocardiogram; however, this test did not show any important cardiac morphological abnormalities with the exception of a mild mitral regurgitation with 62% of an ejection fraction. No thrombi or myocardial wall dyskinesias were identified. The patient was given a provisory transvenous pacemaker (ultrasonically guided), resulting in an immediate improvement of the cardiac symptoms. The levels of myocardial necrosis markers such as troponi and pro-BNP were normal. No signs of an infection, electrolyte disturbances or organic dysfunctions were observed. The C-reactive protein and the erythrocyte sedimentation rates were elevated (Table 1). A cardiac magnetic resonance imaging (MRI) or a myocardial biopsy was not performed due to the recent pacemaker implantation and the risk of hemodynamic instability. An endotracheal intubation was determined to be unnecessary because she was able to protect her airways, even with drowsiness (Glasgow coma score of 13). She recovered a level of consciousness soon after the pacemaker's introduction.

**Table 1:** Laboratory values

	Day 1	Day 3	Day 5	Day 19
CPK (U/L)	20			20
CKMB (ng/mL)	0.32			0.77
Troponin I (ng/mL)	0.022			0.012
Hemoglobin (g/dL)	9.3	9	9.5	9.7
Hematocrit (%)	29.7	28.5	30	32.7
Leukogram (mil/mm)	10,900	8,300	12,000	11,000
Platelets (mil/mm)	682,000	575,000	494,000	648,000
Sodium (mmol/L)	135	138	136	136
Potassium (mmol/L)	4.2	3.2	4.2	4.6
Chloride (mmol/L)	95	100	95	102
Calcium (mmol/L)	2.6	2.3	2.2	2.5
Phosphor (mmol/L)	1.2	1.2	0.9	
Magnesium (mmol/L)	0.7	0.7	1	0.8
C reactive protein (mg/L)	425	263	70.7	34.7
ESR (mm/hour)	120	120	50	30
Creatinine (mg/dL)	0.5	0.5	0.7	0.5
Urea (mg/dL)	9	17	22	40

CPK - creatinofosfoquinase; CKMB - creatine kinase MB isoenzyme; ESR - eritrocyte sendimentation rate.

**Figure 4:**
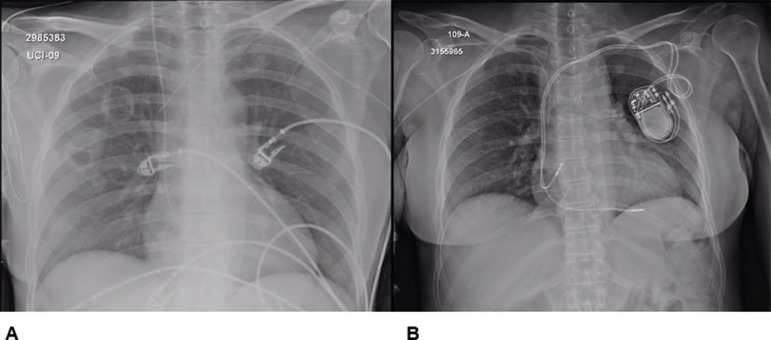
(A) Chest radiography in AP view showing bilateral nodules of the entry on the cardiovascular intensive care unit. (B) Chest radiography in PA view showing location and positioning of the pacemaker.

As the patient recovered hemodynamic stability, she was started on therapy with rituximab at one dose every week for four weeks. After six days, no further episodes of SVT were noted; however, the patient was still dependent on the provisory pacemaker, and a permanent pacemaker was then implanted (Figure 4). The patient was discharged after treatment with broad-spectrum antibiotics due to pneumonia.

**Figure 5:**
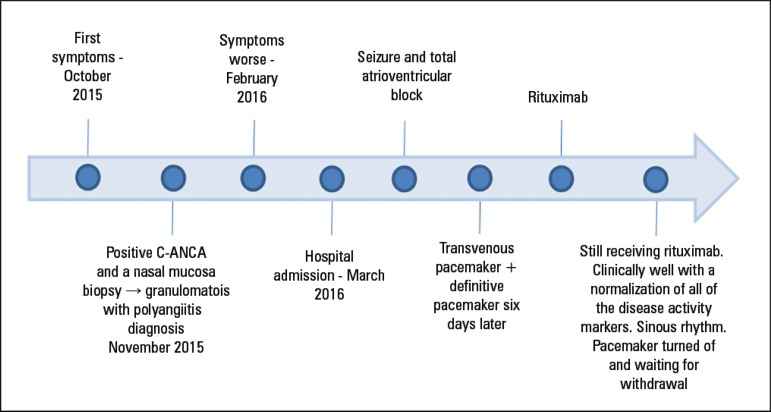
Timeline.

The rituximab treatment was resumed after ambulatory and upper respiratory tract symptoms, as well as general symptoms, improved significantly. Analysis of B lymphocytes using the marker CD19 resulted in near zero levels upon the follow-up visit. After 6 months of immunobiological therapy, normalization of all disease activity markers was observed, and the pacemaker was turned off, as an asymptomatic sinus rhythm was maintained. The patient continues to be followed-up with by the Rheumatology and Cardiology departments, waiting for removal of the permanent pacemaker (Figure 5).

## DISCUSSION

Granulomatosis with polyangiitis is a systemic inflammatory disorder of unknown etiology^(1,3)^ that is characterized by a vasculitis of the small arteries, the arterioles and the capillaries, together with necrotizing granulomatous lesions. Clinical presentation of granulomatosis with polyangiitis depends upon the affected organ and the degree of progression from local to systemic arteritis.^(3)^ The upper and lower respiratory tracts, the kidneys and the eyes are typical sites of occurrence and observed in 50 to 60% of clinical cases.^(1,5)^ In approximately 8% to 16% of the cases, the eyes can be the only site of affliction at the initial presentation, while persistent symptoms are recorded in 87% of all of patients.^(4)^ A positive c-ANCA or tissue biopsy are important for the initial diagnosis^(1)^ in order to exclude other diseases with a similar presentation.

Several types of ANCA can be recognized, but the two subtypes relevant to the onset of systemic vasculitis are those that are directed towards proteinase-3 (PR3) and myeloperoxidase. c-ANCA is related to the PR3 ANCA and has a high specificity (> 90%), which helps in the diagnosis of granulomatosis with polyangiitis and other closely related diseases.^(3)^

Early treatment is crucial in order to prevent severe complications and often to preserve life. For those patients with a severe disease, there are now two co-treatment options for inducing a remission: cyclophosphamide plus corticosteroids or rituximab plus corticosteroids. Remission can be induced in greater than 90% of the patients who are treated with either of these two therapies.^(5)^

Cardiac involvement in granulomatosis with polyangiitis occurs in 6% to 44% of the cases and is secondary to necrotizing vasculitis with granulomatous infiltrates.^(6)^ Pericarditis is the most common cardiac manifestation (35%), followed by cardiomyopathy (30%), coronary artery disease (CAD) (12%), valvar disease (6%), concomitant CAD and valvar disease (6%), concomitant pericarditis and cardiomyopathy (1.6%), and severe conduction disorders (1.6%).^(3)^

Atrial tachycardia, atrial fibrillation and flutter are the most common arrhythmias that are found in patients diagnosed with granulomatosis with poliangiitis. Ventricular arrhythmias are usually noted in association with dilated cardiomyopathy, cardiac ischemia or secondary to cardiac masses and are uncommon in hearts with no structural damage.^(6)^ All conduction defects varying in severity can be recognized, including intraventricular conduction defects, first and second degree heart blocks and a complete heart block. Treatment for these types of heart conduction system dysfunctions may require a transient or a permanent pacemaker depending on whether the arrhythmia is induced by reversible causes such as hydro-electrolytic disturbances or drugs.^(1)^

A bedside echocardiogram may be a valuable tool to assist intensive care physicians with differential diagnoses of shock and to assess structural heart diseases or ventricular dysfunctions in cases of unexplained arrhythmias. In this case, bedside echocardiography testing was useful in determination of shock diagnosis and subsequently the decision of a quickly introduced pacemaker.^(7)^

Only ten reported cases of granulomatosis with poliangiitis patients with a concurrent atrioventricular blockage have been reported within the last ten years.^(8-19)^ What made our patient different was that she presented with SVT, a life threatening condition that can be associated with an atrioventricular block, leading the patient to state of hemodynamic instability. These complications are common in patients with dilated cardiomyopathy, ischemia, and electrolyte disturbances and are secondary to cardiac masses; however, our patient did not have any of the above stated diagnoses or macroscopical cardiac structural disease as determined via echocardiography.

Cardiac MRI and myocardial biopsy would be beneficial in order to diagnose any cardiac involvement of granulomatosis with polyangiitis disease. An MRI was not performed in this case due to the presence of the pacemaker, and a biopsy was not performed due the risks inherent to the procedure. The elevated levels of inflammatory markers, along with clear signs of the disease activity on the day of the heat block and SVT, were associated with no other findings of a structural heart disease, ventricular dysfunction, electrolyte disturbances, or drugs; thus, the diagnosis of granulomatosis with polyangiitis with a cardiac involvement was determined to be the most likely cause. Furthermore, the immunosuppression therapy resulted in complete improvement of the patient, and the pacemaker was turned off and withdrawn from use.

## CONCLUSION

In summary, cardiac involvement with an atrioventricular block is an uncommon complication in the development of granulomatosis with poliangiitis. The diagnosis accounts for significant morbidity, especially when associated with hemodynamic deterioration or ventricular tachyarrhythmias. Early detection and specific intervention are able to prevent unfavorable outcomes, specifically in the intensive care unit.
